# Island Sign Predicts Hematoma Expansion and Poor Outcome After Intracerebral Hemorrhage: A Systematic Review and Meta-Analysis

**DOI:** 10.3389/fneur.2020.00429

**Published:** 2020-06-04

**Authors:** Yufei Wei, Guangming Zhu, Yonghong Gao, Jingling Chang, Hua Zhang, Nan Liu, Chao Tian, Ping Jiang, Ying Gao

**Affiliations:** ^1^Department of Neurology, Dongzhimen Hospital, Beijing University of Chinese Medicine, Beijing, China; ^2^Department of Radiology, School of Medicine, Stanford University, Stanford, CA, United States; ^3^Key Laboratory of Chinese Internal Medicine of Ministry of Education and Beijing, Dongzhimen Hospital Affiliated to Beijing University of Chinese Medicine, Beijing, China; ^4^Department of Neurology, The Seventh Medical Center of PLA General Hospital, Beijing, China; ^5^Institute for Brain Disorders, Beijing University of Chinese Medicine, Beijing, China

**Keywords:** intracerebral hemorrhage, island sign, hematoma expansion, poor outcome, non-contrast computed tomography

## Abstract

**Background:** Early hematoma expansion (HE) occurs in patients with intracerebral hemorrhage (ICH) within the first few hours from ICH onset. Hematoma expansion has been considered as an independent predictor of poor clinical outcome and mortality after ICH. Island sign (IS) on the non-contrast computed tomography (NCCT) appears to increase the rate of detection of HE. However, there is insufficient evidence to declare that IS is an independent predictor for ICH patients prognosis and classification.

**Objectives:** To investigate whether IS on NCCT could predict HE and functional outcome following ICH.

**Methods:** Major databases were systematically searched, including PubMed, EMBASE, Cochrane library, and the Chinese database (CNKI, VIP, and Wanfang databases). Studies about the associations between IS and HE or IS and clinical outcome were included. The pooled result used the odds ratio (OR) with a 95% confidence interval (CI) as effect size. Heterogeneity and publication bias were assessed. Subgroup analysis and meta-regression were applied to detect potential factors of heterogeneity.

**Results:** Eleven studies with 4,310 patients were included in the final analysis. The average incidence rate of IS and HE were 21.58 and 33%, respectively. The ideal timing for assessing HE was also not uniform or standardized. We separately performed two meta-analyses. First, 10 studies were included to estimate the association between IS and HE. The pooled OR was statistically significant (*OR* = 7.61, 95% CI = 3.10–18.67, *P* < 0.001). Second, four studies were included in the meta-analysis, and the pooled result showed that IS had a significantly positive relationship with poor outcome (*OR* = 3.83, 95% CI = 2.51–5.85, *P* < 0.001).

**Conclusions:** This meta-analysis showed that NCCT IS is of great importance and value for evaluation of HE and poor outcome in patients with ICH. Future studies should focus on developing consensus guidelines, and more studies with large sample size and longitudinal design are needed to validate the conclusions.

## Introduction

Intracerebral hemorrhage (ICH), the most devastating subtype of stroke, leads to high mortality and morbidity (1, 2). Early hematoma expansion (HE) is used referring to significant hemorrhage enlargement (>33% and/or >6 mL) and occurs within the first few hours of onset in 38% of ICH patients (3). Hematoma expansion has been considered as an independent predictor of poor clinical outcome and mortality after ICH (4). Early identification of patients at high risk of HE and accurate prognostic evaluation is critical for ICH patients.

Currently, the spot sign on computed tomography angiography (CTA) is recognized as an independent imaging marker with reliable sensitivity and specificity to identify HE (5). However, CTA is not routinely performed because of its high price and being time-consuming. Furthermore, CTA is not readily available in most primary hospitals in developing countries. In contrast, non-contrast computed tomography (NCCT) is a widely available and generally well-tolerated alternative to CTA.

Over the last decade, several NCCT imaging markers have been developed for identification and characterization of HE after ICH, such as hypodensities, blend sign, satellite sign, island sign (IS), and shape irregularity (6–8). Most NCCT imaging features are density-related or shape-related biomarkers. Nevertheless, no consensus exists in diagnostic criteria, and past studies are therefore lacking in clarity and consistency. Recently, Morotti et al. (9) proposed practical guidelines for detecting, interpreting, and reporting these radiological markers, which may provide an additional boost in the prediction performance of NCCT markers.

The NCCT IS, a reliable predictor of experiencing HE and clinical deterioration in patients with ICH, was first reported by Li et al. (10). Island sign is defined as (1) three or more scattered small hematomas all separated from the main hematoma, or (2) four or more small hematomas some or all of which may be connected with the main hematoma. Some investigations have reported that IS can predict the prognosis of ICH (11–14), whereas others have reported that there was no significant correlation between IS and HE (15). There is insufficient evidence to declare that IS is an independent predictor for ICH patients' prognosis and classification. Thus, we first introduced this meta-analysis to assess the accuracy of IS in predicting HE and functional outcome in patients with ICH.

## Materials and Methods

### Search Strategy

This systematic review was performed according to PRISMA (Preferred Reporting Items for Systematic Reviews and Meta-Analysis) statement (16). Two reviewers (YW and YGao) independently completed the literature search through the major databases, including PubMed, EMBASE, Cochrane library, and the Chinese database (CNKI, VIP, and Wanfang databases) from inception to December 2019. Hand searching of Web of Science and Google Scholar identified potential eligible studies. There were no language or date restrictions. MESH (Medical Subject Headings) and free word of “cerebral hemorrhage” and “island sign” and “hematoma expansion” were used in the search strategy (see Appendix 1 in Supplementary Material).

### Study Selection

Records identified on the search were separately screened and assessed for inclusion by two reviewers (CT and PJ). Discrepancies were resolved by discussion or consultation with a third reviewer (GZ). Studies were selected if they met the following criteria: (1) ICH patients aged ≥18 years, (2) investigating IS on NCCT, (3) examined the associations between IS and HE or IS and functional outcome, (4) the functional outcomes were assessed by Glasgow Outcome Scale or modified Rankin Scale at 90 days. Surgical treatment conducted before follow-up CT scans was excluded. Patients were also excluded if they were diagnosed with secondary ICH, including arteriovenous malformation, trauma, aneurysm, tumor, or brain infarction.

### Data Extraction

Two reviewers (NL, JC) independently extracted data and cross-checked to reduce the risk of errors. Extracted data included characteristics of studies (author, year, study design, country the research was done), mechanism of ICH, the incidence and definition of IS and HE, and the definition of functional outcome (Table 1, Appendix 1 in Supplementary Material). The odds ratio (OR) was used as a measure of association between the outcome and exposure. The raw incidence data were used to calculate the unadjusted crude OR. To control for confounding covariates, adjusted OR resulting from the multivariate analysis was selected preferentially.

**Table 1:** Characteristics of included studies.

**References**	**Study population**	**Sample size, male (%)**	**Age (years)**	**Time from onset to CT (h)**	**Time from follow-up CT to diagnostic CT (h)**	**Definition of HE and poor outcome**	**Prevalence of IS (%)**	**Prevalence of HE (%)**	**NOS scores**
Du et al. (17)	China	402, 66.2	Mean, *SD*: 60.2, 14.6	Within 24	Within 24	>6 mL or 33% on follow-up CT	28.1	31.6	8
Huang et al. (18)	China	266, 65.0	Mean, *SD*: 56.0, 10.9	Within 24	Within 24	>6 mL or 33% on follow-up CT	22.9	37.22	8
Law et al. (15)	Malaysia	2078, 56.0	Mean, *SD*: 68.9, 13.8	Within 8	Within 36	>6 mL or 33% on follow-up CT mRS score >3 at day 90	8.9	27.4	8
Li et al. (19)	China	282, 66.3	Mean, *SD*: 60, 12.2	Within 6	Within 36	>6 mL or 33% on follow-up CT mRS score >2 at day 90	14.9	31.2	9
Qin et al. (20)	China	46, 58.7	Mean, *SD*: 62.8, 8.8	Within 6	Within 24	>6 mL or 33% on follow-up CT	30.42	30.43	6
Wang et al. (21)	China	156, 55.1	Mean, *SD*: 64.8, 11.9	Within 6	Within 24	>6 mL or 33% on follow-up CT	30.8	35.26	8
Wang et al. (21)	China	180, 57.4	Mean, *SD*: 61.2,12.7	Within 6	Within 72	>6 mL or 33% on follow-up CT	24.45	34.44	7
Xie et al. (14)	China	251, 67.3	Mean, range: 62.2, 24–93	Within 6	Within 24	>6 mL or 33% on follow-up CT	12.07	40.8	9
Zhang et al. (13)	China	283,70.3	Mean, *SD*: 60.2, 10.7	Within 6	Within 24	>6 mL or 33% on follow-up CT GCS score <3 at day 90	23.3	39.93	9
Zheng et al. (12)	China	165, 72.1	Mean, *SD*: 59.5, 12.0	Within 6	Within 24	>12.5 mL or 33% on follow-up CT	20	24.8	7
Sporns et al. (11)	Germany	201, 54.7	Median, range: 68, 55–79	Within 6	NA	mRS score >3 at day 90	26.4	NA	8

*SD, standard deviation; HE, hematoma expansion; CT, computed tomography; mRS, modified Rankin Scale; GCS, Glasgow Coma Scale; IS, island sign; NOS, Newcastle–Ottawa Scale*.

### Quality Assessment

Quality assessment was independently performed by two reviewers (CT and PJ) with the Newcastle–Ottawa Scale (NOS) (22). The NOS criteria included three aspects: (1) subject selection: 0–4; (2) comparability of subject: 0–2; (3) clinical outcome: 0–3. Newcastle–Ottawa Scale scores ranged from 0 to 9, and a score ≥7 indicates good quality. We resolved discrepancies through discussion, and if necessary, a third reviewer (HZ) was consulted. Quality assessment was applied to detect potential factors of heterogeneity.

### Statistical Analysis

The statistical analysis was performed using Stata Statistical Software (version 15.0; Stata Corp., College Station, TX, USA). We defined the pooled OR with 95% confidence interval (CI) as effect size. Heterogeneity among studies was estimated by the Cochrane's *Q* statistic and *I*^2^ statistic. If *Q* statistic exhibits *P* < 0.1 or *I*^2^ test shows >50%, which indicates significant heterogeneity, the random-effects model was applied for pooling the results, or else the fixed-effects model was used. Subgroup analysis and meta-regression were applied to detect potential factors of heterogeneity. The potential sources of heterogeneity included the following: study design, population, sample size, the time interval from symptom onset to CT, time interval from follow-up CT to diagnostic CT, and NOS scores. Begger test was applied to investigate publication bias.

## Results

### Study Selection

**Figure 1:**
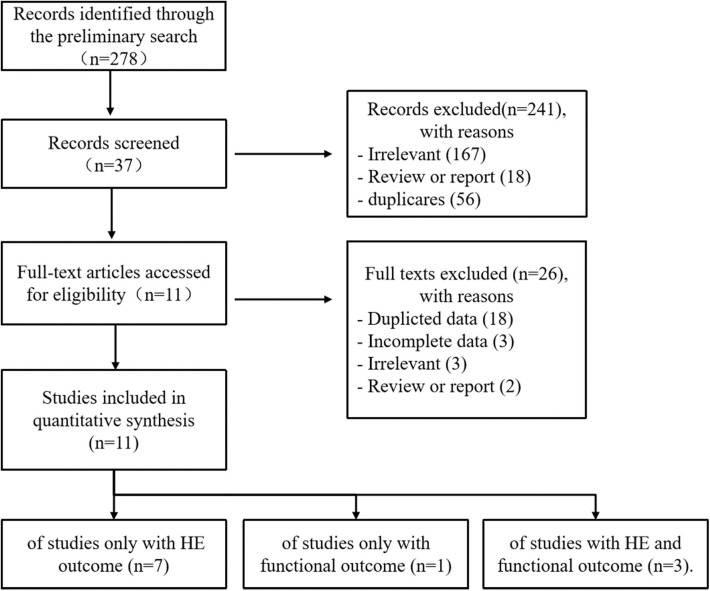
Flowchart of the study selection.

Our study selection process is shown in Figure 1. The preliminary search identified 278 records: PubMed (*n* = 136), EMBASE (*n* = 74), Cochrane Library (*n* = 7), CNKI (*n* = 8), VIP (*n* = 4), Wangfang (*n* = 6), and additional 43 records identified through Web of Science and Google Scholar. After removing duplicates and irrelevant records, the full texts of 39 articles were accessed for eligibility. Two conference discussions and five third-party consultations were organized to resolve discrepancies. Ultimately, 11 studies were included in the meta-analysis. Of these, seven studies reported only HE outcome, one study reported only functional outcome, and three studies reported HE and functional outcome.

### Characteristics of Included Studies

Eleven studies with 4,310 patients were included in the final analysis, including nine prospective studies and two retrospective studies (11–15, 17–21, 23). Overall, nine studies were conducted in China, one in Malaysia, and one in Germany. The sample size of the included studies ranged from 46 to 2,078. The age and sex were balanced between HE groups and non-HE groups in the primary literature. Eight studies determined HE with >6 mL or >33%, and two studies with >12.5 mL or >33%. Time interval from symptom onset to CT ranged from 6 to 24 h. Time interval from follow-up CT scan to initial CT ranged from 24 to 72 h. The average incidence rates of IS and HE were 21.58 and 33%, respectively. Newcastle–Ottawa Scale scores of all included studies ranged from 6 to 9. The detailed description about NOS is shown in Appendix 1 (Supplementary Material).

Nine of 11 studies reported adjusted OR and clinical factors associated with HE, such as age, sex, admission GCS score, hematoma volume, anticoagulant use, and onset-to-CT time. These potential risk factors or confounding variables were included as the risk-adjusting variables. All included studies excluded anticoagulation-related ICH, except studies by Qin et al. (20) and Law et al. (15), in which they enrolled anticoagulation treatment as a confounding factor and performed multivariate logistic regression analyses to exclude the influences of those confounding factors.

### Predictive Value of Island Sign for HE

**Figure 2:**
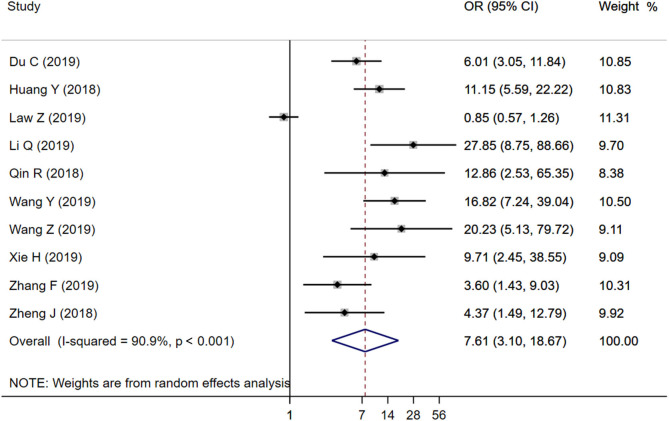
Forest plots of Island Sign on NCCT and HE.

**Figure 3:**
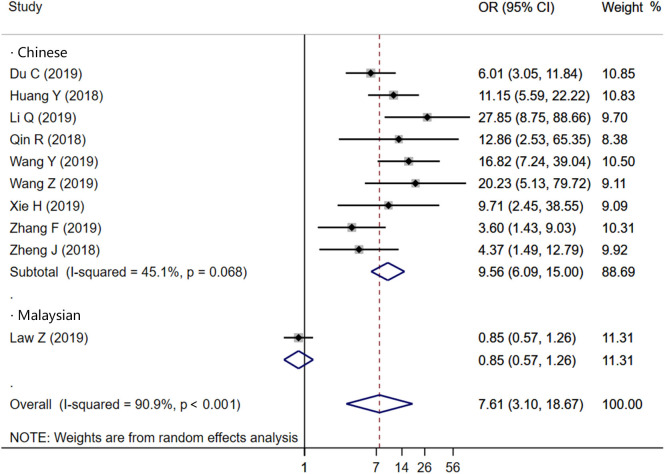
Forest plots of subgroup analysis of Island Sign on NCCT and HE according to population.

Ten studies were included to analyze the association between IS and HE. The random-effects model was conducted because of the existence of significant heterogeneity between studies. The pooled OR was statistically significant (*OR* = 7.61, 95% CI = 3.10–18.67, *P* < 0.001), with substantial heterogeneity (*I*^2^ = 90.9%, *P* < 0.001; Figure 2). The results of our meta-analysis revealed that IS is significantly associated with increased risks of HE. Furthermore, subgroup analysis and meta-regression analysis revealed that the study population was the origin of heterogeneity. Subgroup analysis by populations suggested that the presence of IS might increase the risk of HE among Chinese populations (*OR* = 9.56, 95% CI = 6.09–15.00, *P* = 0.011) with moderate heterogeneity (*I*^2^ = 45.1%, *P* = 0.068; Figure 3), but not among Malaysian populations (*OR* = 0.85, 95% CI = 0.57–1.26, *P* = 0.011; Figure 3). Publication bias was not detected using Begg test (*t* = 0.94, *P* = 0.348).

### Predictive Value of Island Sign for Poor Outcome

**Figure 4:**
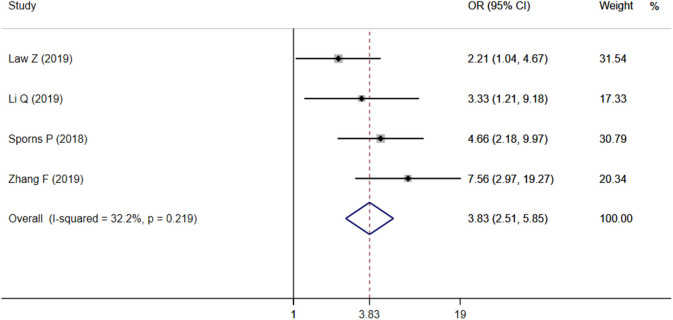
Forest plots of Island Sign on NCCT and poor outcome.

Four studies focused on the relationship between IS and poor outcome, and the pooled OR was statistically significant (*OR* = 3.83, 95% CI = 2.51–5.85, *P* < 0.001), with low heterogeneity (*I*^2^ = 32.2%, *P* = 0.219; Figure 4). The Begg test did not display statistical evidence for publication bias (*t* = 1.70, *P* = 0.089).

## Discussion

The NCTT IS was first proposed for predicting HE by Li et al. (10). Although past research has been conducted on the predictive value of IS, the conclusions have been controversial because of the variety of the diagnostic criteria. In our research, we found that the definition of IS is subjective, which relies on the researcher's personal judgment and lacks objective quantitative indicators. Besides, different diagnostic criteria of HE have been defined across studies, which may account for heterogeneity across studies (9). Most studies determine HE usually with >6 mL or >33%, and some of the studies with >12.5 mL or >33%. Unified diagnostic criteria are the key to predict HE and promote clinical application accurately.

Our meta-analysis results showed that IS is positively associated with the risk of HE (*P* < 0.001), indicating that IS seems to be the optimal shape-related predictor for HE. Importantly, there may be ethnic and geographical variations in the conclusions. The majority of the studies were conducted in China. Therefore, the conclusions are representative of the Chinese population at large, and similar results were not observed among Malaysians (15). The discrepancy may account, at least in part, for the diagnostic criteria. For instance, the ideal timing for assessing HE was also not uniform or standardized. Recently, Morotti et al. (9) proposed practical guidelines for detecting, interpreting, and reporting NCCT predictors of HE, which are expected to improve the accuracy of IS for predicting HE in ICH patients.

This study also found that IS was predictive of poor functional outcome. However, only four studies were eligible for inclusion in the meta-analysis. Thus, there were no sufficient data to make a credible conclusion. Although many researchers have confirmed that early hematoma enlargement closely related to poor outcome, the exact pathophysiological mechanism remains unclear. It was believed in the past that the pathological mechanism of HE was a single persistently bleeding vessel (24). As with the CTA spot sign, multiple ISs appeared around the main hematoma, suggesting hematoma comprised multivessel lesions rather than a single-vessel lesion (25).

A recent meta-analysis summarized that several NCCT imaging signs, such as mixed density, blend sign, black hole, swirl sign, and hypodensity sign, could be considered optimal predictors of HE and neurological outcome (26–29). Among these NCCT predictors, part of definitions overlap. Computed tomography hypodensities, swirl sign, blend sign, and black-hole sign are density-related biomarkers, whereas satellite sign and IS are shape-related biomarkers (28). Because HE is a very complex process involving numerous pathological processes, prediction models with multiple biomarkers or a combination of a single model are imperative. New developments in machine learning and big data may help to promote the development of NCCT imaging signs (30).

Several limitations deserve consideration in the present study. First, most study population came from China, which may not have been sufficiently large to be generalized to other ethnic groups. Moreover, even with the utility of subgroup analysis and meta-regression, the origin of heterogeneity and the pooled result still showed moderate heterogeneity. Finally, the limited number of studies that looked at functional outcomes does not allow us to draw robust conclusions.

## Conclusions

Despite the limitations described above, the NCCT IS is of great importance and value for evaluation of HE and poor outcome in patients with ICH. Besides, the inconsistency among studies may be due to differences in ethnicity and language, as well as discrepancies brought by non-standardized diagnostic criteria. Future studies should focus on developing consensus guidelines, and more studies with large sample size and longitudinal design are needed to validate the conclusions.

## Data Availability Statement

All datasets generated for this study are included in the article/Supplementary Material.

## Author Contributions

YiG designed this study. YW and YoG did the literature search. JC and HZ were responsible for the analysis or interpretation of data. CT, NL, and PJ evaluated the quality of studies. YW wrote the first draft of this manuscript. GZ and YoG did a critical revision of the final manuscript. All authors listed have made a substantial contribution to the work.

## Conflict of Interest

The authors declare that the research was conducted in the absence of any commercial or financial relationships that could be construed as a potential conflict of interest.
