# Supplementary material for: Island Sign Predicts Hematoma Expansion and Poor Outcome After Intracerebral Hemorrhage: A Systematic Review and Meta-Analysis
Source: Front Neurol. 2020 Jun 4;11:429. doi: 10.3389/fneur.2020.00429 (PMC7287172; doi:10.3389/fneur.2020.00429)
Supplement: Supplementary file 1 [file Table_1.DOCX]

**Appendix 1 Search strategies**

EMBASE

#1 'brain hemorrhage'/exp

#2 'cerebral h?emorr*' OR 'intracerebral h?emorr*' OR 'brain h?emorr*' OR 'intraventricular h?emorr*' OR 'hemorrhagic stroke'/exp OR 'hemorrhagic stroke'OR ich

#3 #1 OR #2

#4 'island sign*' OR 'noncontrast ct' OR 'nonenhanced ct' OR ncct

#5 'hematoma enlargement' OR 'hematoma growth' OR 'hematoma expansion'/exp OR 'hematoma expansion'

#6 #3 AND #4 AND #5

PubMed

#1 (((((("cerebral hemorrhage"[Mesh]) OR ((((((Intracerebral h?emorr*) OR Cerebral h?emorr*) OR Brain h?emorr*) OR intraventricular h?emorr*) OR hemorrhagic stroke) OR ICH)))))

#2 (((((Island sign) OR Island signs) OR noncontrast CT) OR nonenhanced CT) OR NCCT))

#3 (((hematoma enlargement) OR hematoma growth) OR hematoma expansion)

#4 #1 AND #2 AND #3

**Supplemental Table 1. Table 1: Characteristics of included studies.**

| Author | Year | the odds ratio of HE  (OR, 95% CI) | the odds ratio of poor outcome  (OR, 95% CI) |
| --- | --- | --- | --- |
| Du C | 2019 | adjusted OR: 6.014 (3.054-11.843) | - |
| Huang Y | 2018 | adjusted OR: 11.15 (5.59-22.22) | - |
| Law Z | 2019 | adjusted OR: 0.85 (0.57-1.26) | adjusted OR: 2.21 (1.04-4.67) |
| Li Q | 2019 | adjusted OR: 27.85 (8.75-88.66) | adjusted OR: 3.33 (1.21-9.18) |
| Qin R | 2018 | unadjusted OR: 12.857 (2.53-65.347) | - |
| Wang Y | 2019 | unadjusted OR: 16.82 (7.245- 39.039) | - |
| Wang Z | 2019 | adjusted OR: 20.23 (5.13-79.72) | - |
| Xie H | 2019 | adjusted OR: 9.712 (2.447-38.547) | - |
| Zhang F | 2019 | adjusted OR: 3.596 (1.432-9.028) | adjusted OR: 7.564 (2.969-19.273) |
| Zheng J | 2018 | adjusted OR: 4.371 (1.493-12.795) | - |
| Sporns P | 2018 | - | adjusted OR:4.66 (2.18-9.97) |

**Supplementary Table 2. Quality scores of included studies using Newcastle-Ottawa Scale**

| Study | Selection | | | | Comparability | outcome | | | |
| --- | --- | --- | --- | --- | --- | --- | --- | --- | --- |
|  | Representativeness of the exposed cohort | Selection of the non-exposed cohort | Ascertainment of exposure factors | Demonstration that HE was not present at start of study | Comparability on the basis of the design or analysis | Assessment of outcome | Adequate follow-up duration (>24 hours) | Adequate follow-up rate (>80%) | Overall quality |
| Du C  (2019) | 1 | 1 | 1 | 1 | 2 | 1 | 1 | 0 | 8 |
| Huang Y  (2018) | 1 | 1 | 1 | 1 | 2 | 1 | 1 | 0 | 8 |
| Law Z  (2019) | 0 | 1 | 1 | 1 | 2 | 1 | 1 | 1 | 8 |
| Li Q  (2019) | 1 | 1 | 1 | 1 | 2 | 1 | 1 | 1 | 9 |
| Qin R  (2018) | 1 | 1 | 0 | 1 | 1 | 1 | 1 | 0 | 6 |
| Wang Y (2019) | 1 | 1 | 1 | 1 | 1 | 1 | 1 | 0 | 8 |
| Wang Z  (2019) | 1 | 1 | 1 | 1 | 1 | 1 | 1 | 0 | 7 |
| Xie H  (2019) | 1 | 1 | 1 | 1 | 2 | 1 | 1 | 1 | 9 |
| Zhang F  (2019) | 1 | 1 | 1 | 1 | 2 | 1 | 1 | 1 | 9 |
| Zheng J (2018) | 1 | 1 | 1 | 1 | 1 | 1 | 1 | 0 | 7 |
| Sporns P  (2018) | 1 | 1 | 1 | 1 | 2 | 1 | 1 | 0 | 8 |
